# Severe Bacterial Infections and Sepsis in Children and Adolescents in São Paulo, Brazil: A Population-Based Time-Trend Study, 2015-2025

**DOI:** 10.7759/cureus.117966

**Published:** 2026-10-15

**Authors:** André Luís Bastos Lima, Juan Silva Martins, Isadora Rita Carvalho, Cauê da Silva Manara, Stella Bernachi Rodrigues, Julia Pinheiro Almeida, Marcella Magliano Marins, Paola Zelada Florence, Ana Clara Fernandes

**Affiliations:** 1 Medicine, Universidade Ceuma, Imperatriz, BRA; 2 Medicine, Faculdade de Saúde Santo Agostinho, Vitória da Conquista, BRA; 3 Medicine, Faculdade de Medicina de Itajubá, Itajubá, BRA; 4 Medicine, Universidade de Santa Cruz do Sul, Santa Cruz do Sul, BRA; 5 Medicine, Centro Universitário São Camilo, São Paulo, BRA; 6 Medicine, Universidade do Oeste Paulista, Presidente Prudente, BRA; 7 Medicine, Universidade Estácio de Sá (UNESA)-Instituto de Educação Médica (IDOMED), Rio de Janeiro, BRA; 8 Medicine, Universidade Franciscana (UFN), Santa Maria, BRA; 9 Medicine, Hospital Universitario Servidores do Estado, Rio de Janeiro, BRA

**Keywords:** adolescent, bacterial infections, child mortality, hospitalization, sepsis

## Abstract

Background: Severe bacterial infections and sepsis are leading causes of pediatric hospitalization and death worldwide, yet recent Brazilian population-based data spanning the COVID-19 pandemic and distinguishing severe bacterial infection broadly from strict septicemia remain scarce.

Objectives: To describe hospitalization trends, mortality, and factors associated with in-hospital death among children and adolescents with severe bacterial infections and sepsis in São Paulo State, Brazil, from 2015 to 2025, comparing the broad cohort with a strict-sepsis subgroup.

Methods: Population-based, repeated cross-sectional study in São Paulo State, Brazil, 2015-2025, using Hospital Information System of the Brazilian Unified Health System (Sistema de Informações Hospitalares do Sistema Único de Saúde, SIH/SUS) records for hospital admissions of individuals aged 28 days to 19 years. The primary exposure was age group (infant, preschool-age, school-age, adolescent); the primary outcome was in-hospital death. Poisson regression estimated the annual trend in the number of admissions. Crude relative risks (RR) were calculated directly from contingency data; adjusted RR (aRR) were obtained by predictive marginal standardization from a multivariable logistic model, with modified Poisson regression (robust variance) as a sensitivity analysis.

Results: Among 553,218 hospitalizations (277,644; 50.2% male), overall case fatality was 1.6% (8765 deaths). Compared with infants (case fatality 1.8%; 3038/170,495), adolescents had over double the risk of death (crude RR 2.16, 95% CI 2.06-2.27), while preschool-age (RR 0.47, 95% CI 0.44-0.50) and school-age children (RR 0.59, 95% CI 0.54-0.63) had lower risk. Male sex carried modestly higher risk (RR 1.16, 95% CI 1.11-1.21), and comorbidity (49,952; 9.0% of admissions) more than tripled risk (RR 3.22, 95% CI 3.07-3.38). The number of admissions declined over the study period (Poisson regression trend ratio 0.96 per year, 95% CI 0.96-0.97), with a pronounced, non-linear drop during the pandemic and partial recovery thereafter. The strict-sepsis subgroup comprised 27,181 admissions (4.9% of the cohort) but had a case fatality of 14.7% (3985 deaths), nine times that of the broad cohort.

Conclusions: Severe bacterial infections impose a substantial, age-concentrated hospital burden among Brazilian children, while strict sepsis, though comparatively uncommon, remains markedly lethal. Distinguishing these entities is important for surveillance and resource planning.

## Introduction

Severe bacterial infections are a major cause of morbidity and mortality in children. Sepsis represents their most severe systemic manifestation, associated with high mortality and substantial use of hospital resources. Recent global estimates indicate that around half of the 50 million people affected by sepsis annually are neonates and children younger than 19 years, with mortality ranging from 1% to 5% for sepsis and 9% to 20% for severe sepsis [1]. Global Burden of Disease estimates indicate that the large majority of sepsis cases and deaths occur in low- and middle-income countries [2]. In Brazil, bacterial sepsis in children and adolescents remains a relevant cause of hospitalization and death [3], and population-based studies using administrative data have reported considerable associated costs and case fatality [4,5]. Estimates derived from administrative health information systems may nonetheless underestimate sepsis burden, as case ascertainment depends heavily on the coding strategy applied [6].

Pediatric sepsis differs clinically and epidemiologically from sepsis in adults, with infants and young children showing greater immunological vulnerability and more rapidly progressive disease [7]. International consensus criteria define pediatric sepsis as life-threatening organ dysfunction resulting from a dysregulated host response to infection [8]. Two operational definitions coexist in the literature: a broad concept (Sepsis-2), defined as a systemic inflammatory response syndrome associated with infection, and a strict concept (Sepsis-3), defined as infection-related organ dysfunction, identifying a smaller but substantially more severe subgroup [8,9]. In Brazil, the SPREAD-PED study demonstrated high mortality among children admitted to pediatric intensive care units [9].

Studies relying exclusively on septicemia codes may underestimate the hospital burden of severe bacterial infections, as coding accuracy varies with the case-ascertainment strategy used [6]. Nationwide administrative and claims-based data constitute an important resource for population-based epidemiological research [10]. In Brazil, the Hospital Information System (Sistema de Informações Hospitalares do Sistema Único de Saúde (SIH/SUS)) similarly allows estimation of the hospital burden of severe bacterial infections and of the narrower subgroup coded as septicemia. Few Brazilian studies have applied this distinction to patients younger than 20 years; the population-based study by Mangia et al. remains one of the few national studies using this design, but it was restricted to 1992-2006 [3]. A single cross-sectional estimate cannot show whether this burden is rising, falling, or was durably disrupted by the COVID-19 pandemic; only a multi-year trend analysis can address that question, which is directly relevant to hospital bed and workforce planning.

Important gaps remain in the epidemiological knowledge of severe bacterial infections and sepsis among children and adolescents in São Paulo State, particularly for the recent period spanning the COVID-19 pandemic. A long-term, population-based time-trend analysis distinguishing severe bacterial infections from this more severe subgroup may inform hospital bed planning, epidemiological surveillance, vaccination strategy, and public policy to reduce child and adolescent mortality.

We aimed to describe the epidemiological profile, temporal trend, and factors associated with in-hospital mortality among hospital admissions of children and adolescents recorded with an International Classification of Diseases, Tenth Revision (ICD-10) code for severe bacterial infection or sepsis in São Paulo State, Brazil, between 2015 and 2025, comparing a broad, administratively defined cohort with a narrower subgroup identified by a principal-diagnosis septicemia code. Because both cohorts are defined by diagnostic coding rather than by clinical confirmation against Sepsis-2 or Sepsis-3 criteria, the terminology used throughout reflects this administrative basis rather than a clinically adjudicated diagnosis.

Framed according to the Population, Exposure, Comparison, and Outcome (PECO) structure, the population comprised children and adolescents aged 28 days to 19 years hospitalized for severe bacterial infection or sepsis in São Paulo State, Brazil, between 2015 and 2025; the exposures of interest were age group, sex, comorbidity, and ICU admission; the comparisons were infants versus older age groups, admissions with versus without comorbidity, and the broad severe-bacterial-infection cohort versus the strict-sepsis subgroup; and the outcome was in-hospital death, with length of stay, hospitalization cost, and ICU admission as secondary outcomes.

## Materials and methods

Study design and setting

This was a population-based, repeated cross-sectional study with an ecological time-trend component, using secondary data on hospitalizations for severe bacterial infections and sepsis among individuals aged 28 days to 19 years, of both sexes, resident in São Paulo State, Brazil, between 2015 and 2025. No sample size calculation was performed; the study included the complete set of hospital admission records meeting the case definition during the study period, constituting a census rather than a sample.

Data sources

Hospitalization data were obtained from the Hospital Information System of the Brazilian Unified Health System (Sistema de Informações Hospitalares do Sistema Único de Saúde, SIH/SUS), using Hospital Admission Authorization microdata retrieved with the microdatasus R package. Population denominators were obtained from official Brazilian Institute of Geography and Statistics (IBGE) projections. Because SIH/SUS captures only admissions billed to the public Unified Health System, hospitalizations covered exclusively by private health insurance and not reimbursed through SUS are not captured; this is noted as a limitation.

Cohort selection

The study population comprised admissions with a qualifying code for severe bacterial infection or sepsis recorded in the principal diagnosis field or in any of the nine secondary-diagnosis fields of the Hospital Admission Authorization; this is an administrative, coding-based case definition rather than a clinical diagnosis, and because a qualifying code could appear in any of the ten diagnosis fields, the principal diagnosis recorded for a given admission does not necessarily correspond to the qualifying infectious condition--for example, an admission could be coded with a non-infectious principal diagnosis (e.g., a neoplasm) and a qualifying infectious or septicemia code only as a secondary diagnosis. This is the reason non-infectious ICD-10 chapters (e.g., neoplasms, congenital malformations, diseases of the circulatory system) appear among the principal-diagnosis chapters reported in the Results, which tabulate admissions by their principal diagnosis irrespective of which diagnosis field contained the qualifying code. The broad cohort included septicemia codes (A40, A41, A02.1, A22.7, A26.7, A28.2, B37.7) and codes for severe bacterial infections frequently associated with pediatric sepsis: bacterial pneumonia (J13-J15, J18), bacterial meningitis (G00, G03), urinary tract infection (N39.0), skin and soft-tissue infection (L08.0, L08.8), septic arthritis and osteomyelitis (M00, M86), and peritonitis (K65); this code list is exhaustive, and no other ICD-10 codes were used to define cohort membership. A strict-sepsis subgroup was defined, conservatively, by septicemia codes recorded specifically as the principal diagnosis rather than in any diagnosis field; this administrative proxy was chosen to approximate, as closely as the coding structure allows, admissions for which septicemia was the primary reason for hospitalization, but it remains a diagnostic-coding construct and should not be equated with clinically adjudicated pediatric sepsis or septic shock meeting Sepsis-2 or Sepsis-3 organ-dysfunction criteria, which cannot be ascertained from SIH/SUS data. Neonates younger than 28 days were excluded, as neonatal sepsis constitutes a distinct clinical entity. Across the 11 annual SIH/SUS extracts (2015-2025), 4,896,220 hospital admission records among children and adolescents were initially retrieved. Applying the age criterion (28 days to 19 years) excluded 785,111 records, leaving 4,111,109 age-eligible records; applying the case-definition criterion (no qualifying diagnosis code in any of the 10 diagnosis fields) excluded a further 3,557,891 records, leaving 553,218 admissions (11.3% of the raw total) eligible for analysis.

Exposures

The primary exposure for the mortality analysis was age group (infant, 28 days to <1 year; preschool-age, 1-4 years; school-age, 5-9 years; adolescent, 10-19 years), with infants as the reference category. Secondary exposures were sex, presence of comorbidity, and ICU admission; ICU admission is interpreted as a marker of clinical severity rather than a modifiable exposure. Comorbidities were identified from any diagnosis field and comprised neoplasms, immunodeficiency, sickle cell disease, congenital heart disease, prematurity, malnutrition, diabetes mellitus, neurological conditions, chronic respiratory conditions, and organ transplantation; these categories are not mutually exclusive.

Outcomes

The primary outcome was in-hospital death, defined as death recorded in the discharge-outcome field of the Hospital Admission Authorization prior to hospital discharge. Secondary outcomes were length of stay, hospitalization cost, and ICU admission.

Statistical analysis

The temporal trend in the number of admissions was assessed by Poisson regression, estimating the annual trend ratio with 95% CI; no offset for population size was used, so this trend describes the number of admissions rather than a population-standardized incidence rate. This single linear trend ratio was prespecified as a parsimonious summary measure of the overall direction and average annual magnitude of change across the full 11-year period; it is not intended to characterize the shape of the trend, which was instead examined descriptively and graphically in the Results section, given the visually evident non-linear disruption associated with the COVID-19 pandemic, rather than modeled with additional piecewise or interaction terms. Admission rates per 100,000 population, which are population-standardized, were calculated separately using IBGE five-year age strata. Categorical variables are reported as frequencies; continuous variables are reported as medians with interquartile range (IQR) for non-normal distributions. P-values are not reported for group comparisons other than the trend test, consistent with journal policy.

Factors associated with in-hospital death were assessed by multivariable logistic regression (age group, sex, ICU admission, comorbidity; infants as reference). Because logistic regression yields odds ratios, which can diverge materially from relative risk (RR) when the outcome is not rare (up to 14.7% in the strict-sepsis subgroup), effect measures were re-expressed as RR. Crude RRs were calculated directly from the observed contingency tables using the standard delta-method variance of the log risk ratio. Adjusted RR (aRR) were obtained by predictive marginal standardization from the fitted logistic model: for each exposure level, model-based counterfactual risks were predicted for every admission with the exposure set to that level and all other covariates held at their observed values, then averaged across the cohort; 95% CIs were derived from 1,000 parametric simulations drawn from the multivariate normal distribution of the model's estimated coefficients. As a sensitivity analysis, aRR were also estimated by refitting a modified Poisson regression model with robust (sandwich) variance; the two approaches produced closely concordant estimates for every factor and were consistent with an approximate conversion of the original odds ratios (Zhang-Yu method) [11,12].

Analyses were performed using R Statistical Software, version 4.5.2 (R Foundation for Statistical Computing, Vienna, Austria). A two-sided p < 0.05 was considered statistically significant.

Missing data

Missingness was 0% for every variable used in the primary and adjusted mortality analyses; a complete-case analysis was therefore used throughout, and no imputation was required. Only the secondary-diagnosis fields, used solely for the descriptive analysis of secondary diagnoses, had substantial missingness (38.9% of admissions had at least one secondary diagnosis recorded); this does not affect the primary mortality analyses.

Sensitivity analyses

Of 4,896,220 records retrieved, 785,111 (16.0%) were excluded by the age criterion and 3,557,891 (72.7%) by the case-definition criterion; both criteria are structural, defined a priori. As the cohort comprises the complete set of eligible SIH/SUS records meeting the case definition, sampling-related selection bias beyond diagnostic coding accuracy is unlikely, though differential under-coding of sepsis relative to other severe bacterial infections could bias comparisons between subgroups. Applying the E-value method [13], the crude RR for comorbidity (3.22, 95% CI 3.07-3.38) would require an unmeasured confounder associated with both comorbidity and death by a risk ratio of at least 5.9 to fully explain the association; for the adolescent-versus-infant RR (2.16, 95% CI 2.06-2.27), the corresponding E-value is 3.7. Because individual-level income or education data are not captured in SIH/SUS records, each admission was linked, via the patient's municipality of residence, to the 2010 Municipal Human Development Index (IDHM; Atlas Brasil, UNDP), categorized into tertiles (T1, greatest deprivation, to T3, least deprivation); linkage coverage was complete, and the multivariable model was refitted with this covariate added.

Ethics approval

This study used exclusively publicly available, de-identified national administrative data and was therefore exempted from review by a Research Ethics Committee, in accordance with Resolution 510/2016 of the Brazilian National Health Council.

Data and code availability

The data supporting this study are publicly available from SIH/SUS, retrievable with the R package microdatasus; population denominators are publicly available from IBGE. The R code used for data cleaning and analysis is available from the corresponding author upon reasonable request.

## Results

Cohort overview

Between 2015 and 2025, 553,218 hospitalizations for severe bacterial infections and sepsis were identified among children and adolescents aged 28 days to 19 years in São Paulo State (Figure 1). The population was slightly predominantly male (277,644; 50.2%). Preschool-age children were the most affected group (222,667; 40.3%), followed by infants (170,495; 30.8%), school-age children (81,978; 14.8%), and adolescents (78,078; 14.1%).

**Figure 1:**
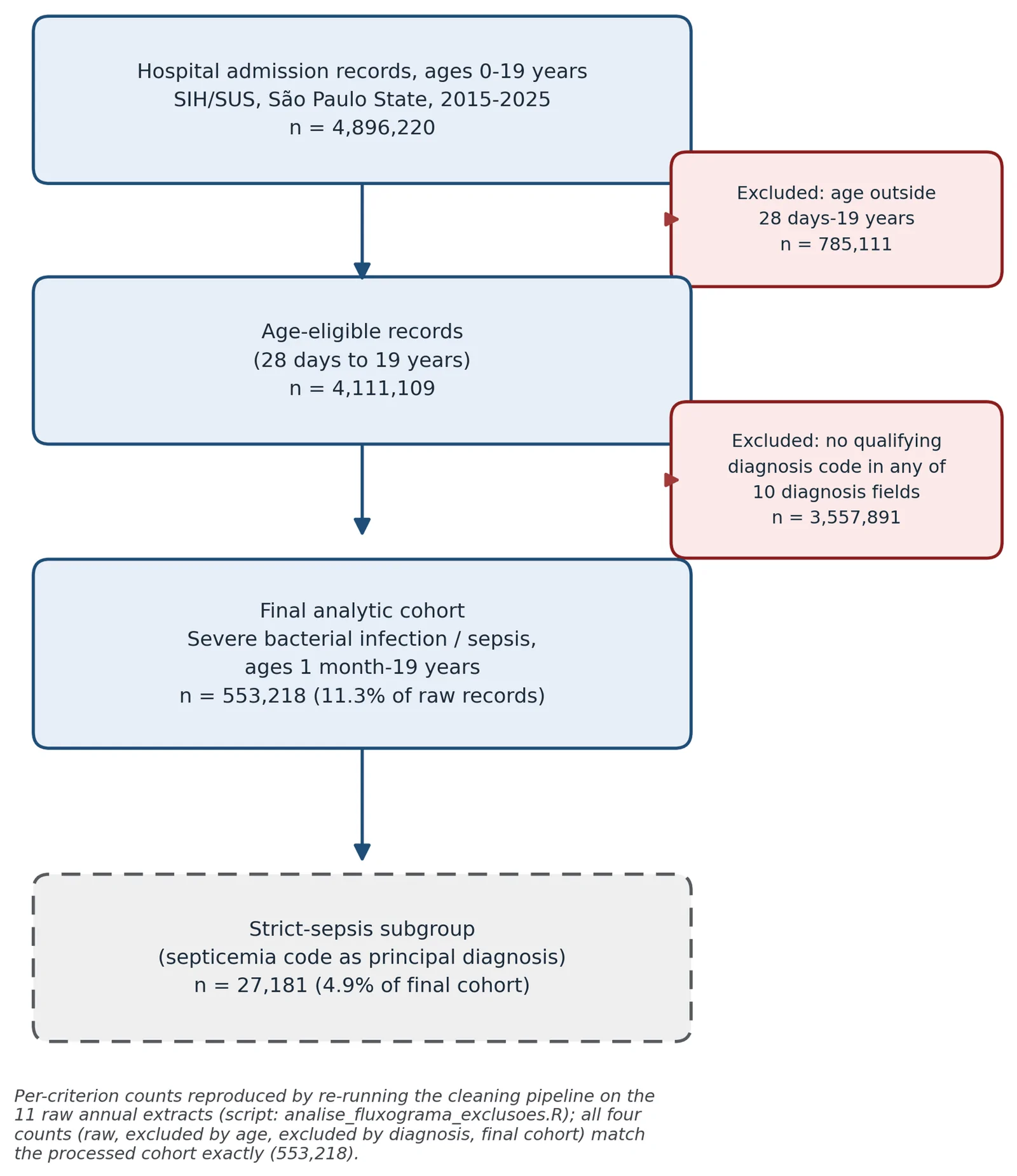
Flow diagram of cohort selection, São Paulo, 2015-2025. All counts (raw records; excluded by age; excluded by case-definition; final analytic cohort) were reproduced exactly by re-running the exclusion pipeline on the 11 raw annual extracts. SIH/SUS: Sistema de Informações Hospitalares do Sistema Único de Saúde.

Temporal trend

The number of admissions declined over the study period (Poisson regression trend ratio 0.96 per year, 95% CI 0.96-0.97; p<0.001), an average annual decrease of approximately 3.5%. However, this average summarizes a markedly non-linear pattern: admissions fell steadily from 2015 to 2019, dropped sharply in 2020 and 2021, and then plateaued at about 50,000 per year from 2022 through 2024 before declining again in 2025, without returning to pre-pandemic levels (Figure 2). Admission rates, standardized by population and therefore comparable across age groups, showed a strong age gradient: children aged 0-4 years had a mean rate of 1182.5 per 100,000, approximately five times that of children aged 5-9 years (246.8) and about 10 times that of adolescents (107.6-122.6). The overall rate fell from 493.1 per 100,000 in 2015 to a nadir of 209.6 in 2020, recovering to 391.7 in 2025.

**Figure 2:**
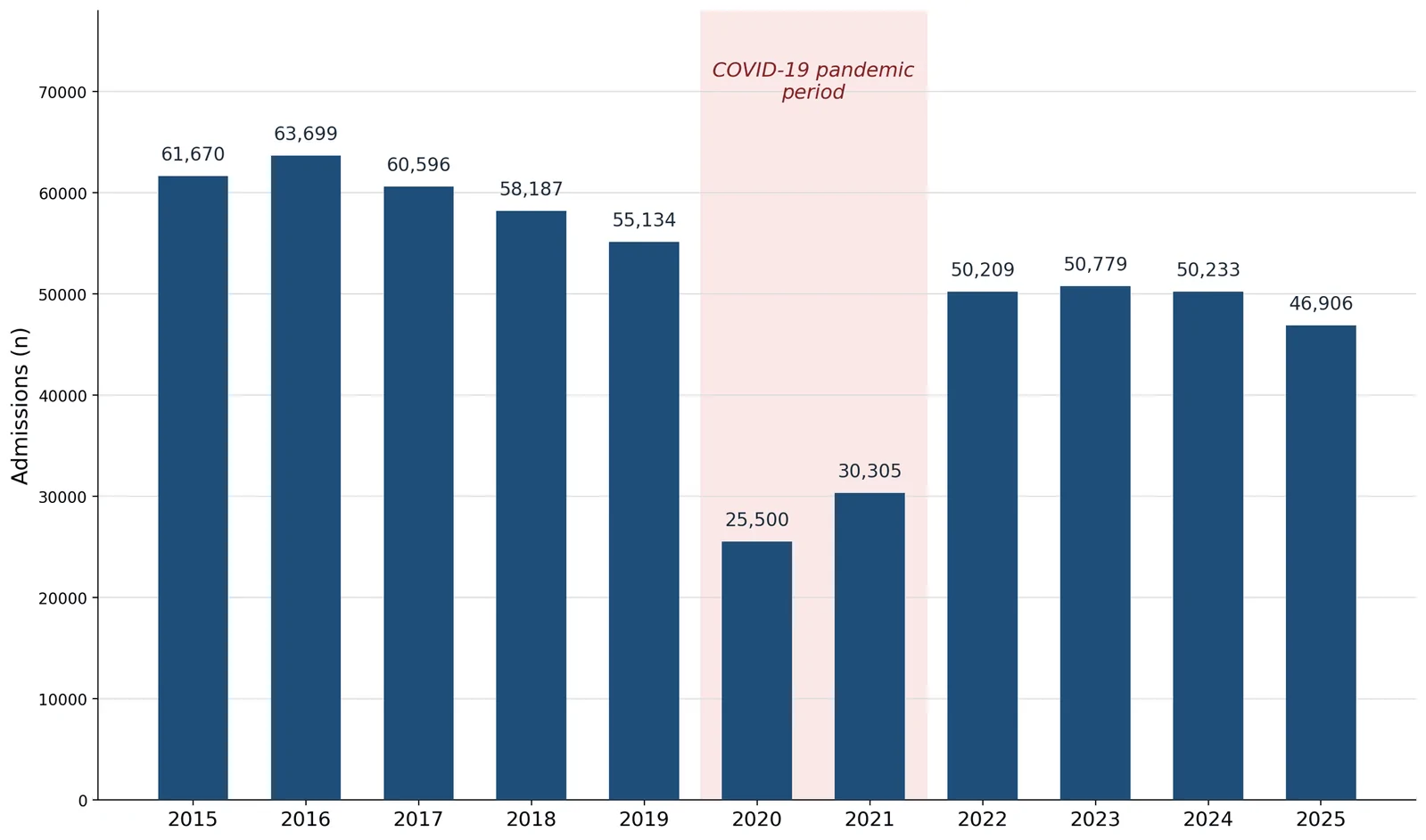
Annual hospitalizations for severe bacterial infections and sepsis among children and adolescents aged 1 month to 19 years, São Paulo State, 2015-2025. Counts are reproduced exactly from the cohort-selection pipeline (Figure 1) and sum to the full analytic cohort (553,218). Corresponding admission rates per 100,000 population are calculated using IBGE population denominators as described in Materials and Methods and are not repeated graphically here. IBGE: Institute of Geography and Statistics.

Clinical characterization

The overall median length of stay was four days. Infants had the longest stay (median five days, IQR 3-8), while preschool-age, school-age, and adolescent groups all had a median of four days, with adolescents showing the widest dispersion (IQR 2-8). Median cost was similar across groups (approximately R$620-630), again with the widest dispersion among adolescents (IQR R$340-1166). ICU admission occurred in 63,669 admissions overall (11.5% of the cohort) and was highest among infants (16.2%) and adolescents (14.2%), and lowest among preschool-age children (7.9%) and school-age children (8.8%).

Admission diagnosis

The most frequent admission-diagnosis chapter was diseases of the respiratory system (407,618 admissions, 2486 deaths; case fatality 0.6%), followed by the genitourinary system (70,641 admissions, 120 deaths; 0.2%), both low-severity. The systemic infectious and parasitic diseases chapter, though smaller (32,358 admissions), concentrated the greatest severity (4287 deaths; case fatality 13.3%; mean length of stay 16.7 days; mean cost R$6596). Similarly elevated case fatality was seen for diseases of the circulatory system (960 admissions, 146 deaths; 15.2%), neoplasms (2065 admissions, 299 deaths; 14.5%), and congenital malformations (1734 admissions, 215 deaths; 12.4%). Diarrheal and intestinal infections were uncommon (1224 admissions, 15 deaths; case fatality 1.2%) (Table 1). Secondary diagnoses were predominantly non-specific respiratory and symptom codes; only 38.9% of admissions had a secondary diagnosis recorded (Table 2).

**Table 1:** Admissions for severe bacterial infections and sepsis by ICD-10 chapter of the admission diagnosis, with case fatality, length of stay, and cost. Length of stay and cost are means. Rows shown are the ICD-10 chapters with the highest admission volume or case fatality and do not sum to the full cohort (n = 553,218); additional, lower-volume chapters (e.g., perinatal conditions, injury, and external causes) are not shown separately. ICD-10: International Classification of Diseases, Tenth Revision.

ICD-10 chapter (admission diagnosis)	n	Deaths	Case fatality (%)	Length of stay (days)	Mean cost (R$)
Respiratory system	407,618	2486	0.6	5.8	1206.83
Genitourinary system	70,641	120	0.2	4.6	513.74
Other infectious and parasitic diseases	32,358	4287	13.3	16.7	6596.16
Digestive system	8460	373	4.4	8.8	3505.12
Nervous system	8246	342	4.2	12.0	3386.28
Musculoskeletal system	7254	27	0.4	8.0	957.31
Skin and subcutaneous tissue	3887	19	0.5	6.0	784.21
Neoplasms	2065	299	14.5	14.3	6907.66
Congenital malformations	1734	215	12.4	22.1	14107.85
Circulatory system	960	146	15.2	16.9	6520.19
Diarrheal and intestinal infections (A00-A09)	1224	15	1.2	5.8	886.67

**Table 2:** Most frequent secondary diagnoses recorded in admissions for severe bacterial infections and sepsis. Percentages are calculated over the total cohort (553,218 admissions). Only 38.9% of admissions had at least one secondary diagnosis recorded, which explains the low individual percentages. ICD-10: International Classification of Diseases, Tenth Revision.

Secondary diagnosis (ICD-10)	n	% of cohort
Fever, unspecified (R50.9)	27,730	5.0
Asthma, unspecified (J45.9)	20,576	3.7
Cough (R05)	20,374	3.7
Bronchopneumonia, unspecified (J18.0)	17,491	3.2
Acute bronchiolitis, unspecified (J21.9)	17,166	3.1
Pneumonia, unspecified (J18.9)	14,993	2.7
Urinary tract infection, site not specified (N39.0)	14,925	2.7
Acute respiratory failure (J96.0)	13,426	2.4
Acute bronchitis, unspecified (J20.9)	11,183	2.0
Dyspnea (R06.0)	10,205	1.8

Mortality and associated factors

Overall case fatality was 1.6% (8765 deaths), and varied by age group: 1.8% (3038 deaths) in infants, 0.8% (1866 deaths) in preschool-age, 1.0% (856 deaths) in school-age, and 3.8% (3005 deaths) in adolescents. Compared with infants, adolescents had more than double the crude risk of death (RR 2.16, 95% CI 2.06-2.27), while preschool-age (RR 0.47, 95% CI 0.44-0.50) and school-age children (RR 0.59, 95% CI 0.54-0.63) had lower risk. Male sex carried a modestly higher crude risk than female sex (1.7% (4718 deaths) versus 1.5% (4047 deaths); RR 1.16, 95% CI 1.11-1.21). Comorbidity, present in 49,952 admissions (9.0%), was associated with a substantially higher crude risk of death (4.3% (2123 deaths) versus 1.3% (6642 deaths) without comorbidity; RR 3.22, 95% CI 3.07-3.38) (Table 3).

**Table 3:** In-hospital mortality, crude relative risk (RR), and adjusted relative risk (aRR) for death. Adjusted RR obtained by predictive marginal standardization from a multivariable logistic model (age group, sex, ICU admission, and comorbidity, mutually adjusted); 95% CI from 1000 parametric simulations of the model's coefficient covariance matrix. A modified Poisson regression sensitivity analysis (robust variance) produced closely concordant estimates: preschool-age 0.71 (0.67-0.76); school-age 0.81 (0.75-0.87); adolescent 2.29 (2.18-2.40); male sex 1.08 (1.03-1.12); comorbidity 1.78 (1.69-1.86); ICU admission 14.09 (13.44-14.78).

Factor	n	Deaths	Case fatality (%)	Crude RR (95% CI)	aRR (95% CI)
Infant	170,495	3038	1.8	1.00 (Reference)	1.00 (Reference)
Preschool-age	222,667	1866	0.8	0.47 (0.44-0.50)	0.72 (0.68-0.76)
School-age	81,978	856	1.0	0.59 (0.54-0.63)	0.81 (0.76-0.87)
Adolescent	78,078	3005	3.8	2.16 (2.06-2.27)	2.30 (2.20-2.41)
Female	275,574	4047	1.5	1.00 (Reference)	1.00 (Reference)
Male	277,644	4718	1.7	1.16 (1.11-1.21)	1.08 (1.03-1.13)
No comorbidity	503,266	6642	1.3	1.00 (Reference)	1.00 (Reference)
Comorbidity	49,952	2123	4.3	3.22 (3.07-3.38)	1.80 (1.72-1.88)
No ICU admission	489,549	2760	0.6	1.00 (Reference)	1.00 (Reference)
ICU admission	63,669	6005	9.4	15.73 (15.01-16.50)	14.13 (13.53-14.80)

Adjusted RR, obtained by predictive marginal standardization, were 2.30 (95% CI 2.20-2.41) for adolescents, 0.72 (0.68-0.76) for preschool-age, and 0.81 (0.76-0.87) for school-age (all versus infants), 1.08 (1.03-1.13) for male sex, and 1.80 (1.72-1.88) for comorbidity. A modified Poisson regression sensitivity analysis produced closely concordant estimates for every factor. Among the factors examined, ICU admission showed by far the strongest association with death, with an adjusted RR of 14.13 (95% CI 13.53-14.80) by marginal standardization and 14.09 (13.44-14.78) by modified Poisson regression, both substantially smaller than the crude odds ratio from the original model (15.73), as expected given that ICU admission is common (11%-16% across age groups) rather than rare (Table 3, Figure 3).

**Figure 3:**
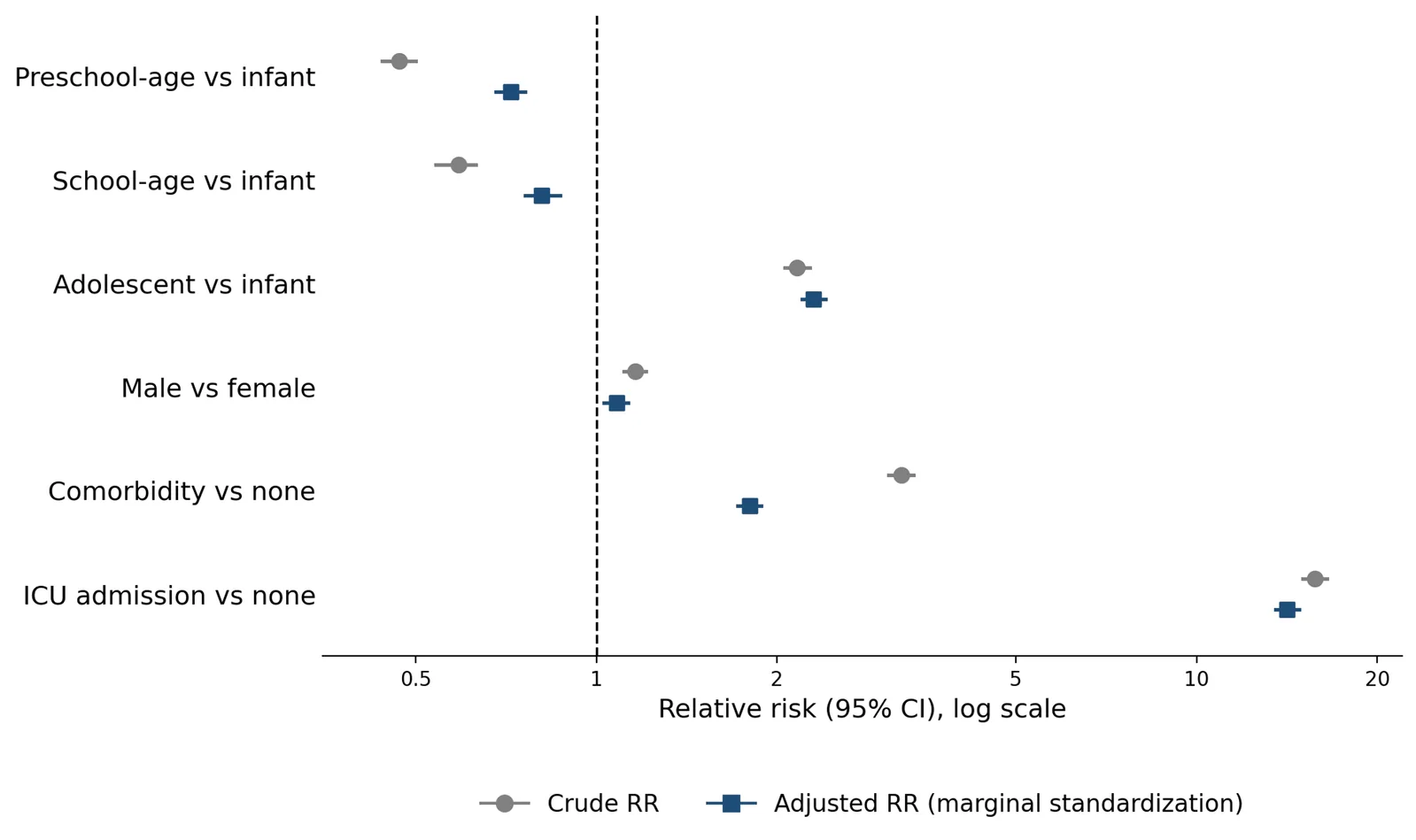
Crude and adjusted relative risk (RR) of in-hospital death, by factor, São Paulo State, 2015-2025. Adjusted RR obtained by predictive marginal standardization from the multivariable logistic model (age group, sex, ICU admission, and comorbidity, mutually adjusted); reference categories (infant age group, female sex, no comorbidity, no ICU admission) omitted, as their RR is 1.00 by definition. Horizontal lines represent 95% confidence intervals; note the logarithmic x-axis.

Strict-sepsis subgroup

The strict-sepsis subgroup (septicemia codes as principal diagnosis) comprised 27,181 admissions (4.9% of the cohort) and had a markedly higher case fatality than the broad cohort (14.7% (3985 deaths) versus 1.6% (8765 deaths); approximately nine-fold higher). Its admission rate was approximately 20 per 100,000 per year, far lower than that of the broad cohort. Unlike the broad cohort, strict-sepsis case fatality remained high across all age groups (12.3% (1473/11,967) in infants, 14.1% (791/5595) in preschool-age, 14.0% (380/2721) in school-age, and 19.4% (1341/6898) in adolescents), without the lower-risk pattern seen in the broad cohort among preschool- and school-age children. Among infants with strict sepsis, the median length of stay reached 17 days and ICU admission occurred in 65.5% of admissions, versus 44%-47% in other age groups.

## Discussion

Principal findings

This population-based study of 553,218 hospitalizations found that severe bacterial infections impose a substantial, age-concentrated burden in São Paulo State, with admissions declining over the study period but remaining highest in early childhood. Mortality followed a distinct U-shaped pattern by age, highest among infants and adolescents. The strict-sepsis subgroup, though comprising under 5% of admissions, accounted for disproportionate mortality, with case fatality roughly nine times that of the broader cohort.

Strengths of the study

This study's principal strengths are its large population-based sample (553,218 admissions), an 11-year observation period spanning the COVID-19 pandemic, and the explicit distinction between the broad cohort of severe bacterial infections and the strict-sepsis subgroup, which is rarely made in Brazilian population-based research. The use of robust methods, including Poisson regression for temporal trends and relative risk rather than odds ratios for the main effect measures, strengthens comparability with the international pediatric epidemiology literature. Unlike most comparable administrative-data cohorts, which adjust only for clinical and demographic factors, we additionally incorporated an area-level socioeconomic deprivation covariate; its addition left the aRR for age group, sex, comorbidity, and ICU admission materially unchanged, providing empirical evidence that socioeconomic confounding does not materially distort the primary associations.

Limitations of the data

Several features of the design should guide interpretation. First, the unit of analysis is the hospital admission, not the individual patient; children with more than one qualifying admission contribute more than once to all estimates, which may result in double-counting from readmissions. The publicly available, de-identified SIH/SUS extracts used in this study do not include a persistent patient-level identifier linking admissions across the 11 annual files, so repeated hospitalizations by the same child could not be identified or statistically clustered; consequently, standard errors were not adjusted for potential within-patient correlation, and the mortality estimates reported here should be interpreted as describing hospital admissions rather than individual patients. Second, this is a repeated cross-sectional, ecological analysis, not a prospective individual-level cohort; therefore, it is descriptive, and the reported RR should not be interpreted causally. Third, SIH/SUS captures only hospitalizations billed to the public Unified Health System; admissions covered exclusively by private health insurance, more common in a wealthier state such as São Paulo, are not captured, which may underestimate the true burden and limit generalizability to settings with a different public-private care mix. Fourth, the study relies on ICD-coded secondary data without clinical confirmation against Sepsis-3 criteria, consistent with evidence that the diagnostic sensitivity of sepsis ICD codes varies widely (5.9%-82.3%), and that agreement between SIH-recorded diagnoses and medical records is low to moderate [14]. This limitation applies to both cohorts examined: the broad severe-bacterial-infection cohort may include admissions in which the qualifying code reflects a less specific or less severe presentation than true severe bacterial infection, and the strict-sepsis subgroup, despite being restricted to a principal-diagnosis septicemia code, remains an administrative proxy rather than a clinically adjudicated diagnosis of sepsis or septic shock meeting Sepsis-2 or Sepsis-3 criteria; neither cohort should therefore be assumed directly comparable to clinically defined pediatric sepsis cohorts, and findings should be interpreted as describing coded hospitalizations rather than clinically confirmed disease. Only 38.9% of admissions had secondary diagnoses recorded, suggesting under-registration of comorbidities and infectious foci beyond what is captured by the structured comorbidity flags used in the adjusted analysis, for which missingness was negligible. Fifth, the close agreement between the marginal standardization and modified Poisson regression estimates supports the robustness of the model specification, though residual confounding by unmeasured factors remains possible. Sixth, SIH/SUS records do not capture individual-level income, education, or household socioeconomic status; the area-level deprivation index used as a partial mitigation is an ecological proxy dated to the 2010 Census, preceding the study period. Seventh, ICU admission was entered as a covariate in the same multivariable model as age group, sex, and comorbidity; because ICU admission may lie on the causal pathway between these factors and death rather than acting solely as a confounder, its inclusion could represent an instance of over-adjustment for a mediator, potentially attenuating the estimated total effect of age, sex, and comorbidity on mortality risk. The adjusted estimates for these factors are therefore best interpreted as effects conditional on severity at the point of ICU triage, rather than as unbiased estimates of their total effect on death. Eighth, the multivariable model assumes that the associations between age group, sex, comorbidity, ICU admission, and in-hospital death were stable across the 11-year study period; this assumption was not formally tested with a calendar-time interaction term, and because the COVID-19 pandemic constituted a documented disruption to case-mix and care-seeking behavior during 2020-2021, residual time-varying confounding of these associations cannot be excluded. Finally, these findings describe hospitalizations financed by the public health system in a single, relatively wealthy Brazilian state; they may not generalize directly to Brazilian regions with different SUS infrastructure, access, or case-mix, or to health systems outside Brazil.

Interpretation

The decline in admissions during 2020-2021, followed by partial recovery, mirrors patterns described in large international cohorts, in which pediatric hospitalizations for infectious disease fell sharply during non-pharmaceutical COVID-19 control measures and rose again after their relaxation [15]. A retrospective multicentric study of hospitalized children in Lebanon spanning the pre-pandemic to pandemic transition similarly documented a shift in the pattern of pediatric respiratory infections across this period [16]. This convergence across different health systems suggests that the transient reduction we observed reflected changes in pathogen transmission dynamics rather than intrinsic changes in disease natural history. The persistently high concentration of admissions among children under 5 years, however, indicates that the pandemic represented only a temporary interruption of a previously established pattern driven by the high baseline incidence of pneumonia, urinary tract infection, meningitis, and other invasive bacterial infections typical of early childhood. Because this study captured the full spectrum of severe bacterial infections, not only sepsis, the high admission rate in early childhood reflects the burden of a broader set of infectious diseases rather than high sepsis incidence per se.

The concentration of mortality, length of stay, and intensive care use at the extremes of the pediatric age range is consistent with classic and contemporary international cohorts describing a similar age-related gradient in severe pediatric infection and sepsis [17-19], and with the well-described immunological vulnerability of infants, particularly those born preterm, who depend heavily on passively transferred maternal antibodies [20]. A comparable age-stratified pattern of resource use has been described for other causes of unplanned pediatric hospitalization in a national administrative database, where readmission risk varied substantially by age and diagnosis rather than uniformly across childhood [21]. The comparatively under-explored elevation of risk among adolescents may reflect that hospitalized adolescents represent a more selected, clinically complex subgroup, with later presentation and greater comorbidity burden, although delayed presentation and detailed clinical complexity were not directly measured in this administrative dataset, and this explanation remains speculative; comorbidity was associated with a more than three-fold increase in crude risk of death, particularly among children with neoplasms, congenital heart disease, and neurological conditions, consistent with previous pediatric intensive-care sepsis cohorts [22].

The nine-fold higher case fatality in the strict-sepsis subgroup compared with the broad cohort is consistent with international evidence that sepsis case-fatality estimates depend heavily on the operational definition applied, with mortality ranging from 13.6% to 24.1% across coding strategies [23], and that explicit septicemia codes identify fewer, more severe cases than broader implicit definitions [24]. A similarly designed national database analysis of infant hospitalizations for respiratory syncytial virus bronchiolitis over more than two decades found that admission rates remained comparatively stable even as markers of disease severity increased and mortality declined [25], illustrating that trend analyses of pediatric infectious hospitalization can reveal discordant trajectories between admission volume and outcome severity, a pattern relevant to our own finding that the burden of severe bacterial infections and sepsis did not decline uniformly across the study period. Sepsis studies should therefore explicitly state the operational definition used, since this choice substantially alters both reported incidence and severity [26]. The high mortality in the strict-sepsis subgroup also occurs against a backdrop of rising antimicrobial resistance among Gram-negative organisms frequently implicated in severe pediatric sepsis in resource-limited settings [27]. The updated 2026 Surviving Sepsis Campaign pediatric guidelines reinforce the importance of early recognition and standardized criteria in reducing preventable mortality [7], which is particularly relevant given the persistently high, age-independent case fatality observed within our strict-sepsis subgroup.

These findings indicate that resource consumption does not scale linearly with admission frequency: planning based solely on admission volume may underestimate demand for high-complexity care, and the sizing of pediatric beds, intensive care units, and specialized teams should incorporate the age-related distribution of severity revealed here.

## Conclusions

The distinction between severe bacterial infection and strict sepsis highlights the contrast between the high care burden of the former and the greater severity and lethality of the latter. The concentration of the most severe outcomes among infants and adolescents reinforces the need for strategies targeting early recognition, organization of the pediatric hospital network, and improved diagnostic coding; because vaccination coverage and other specific preventive interventions were not directly examined in this study, their potential contribution to reducing this burden, while plausible, should be confirmed by studies designed to evaluate them directly. This population-based approach provides a more comprehensive assessment of the burden of these conditions and offers evidence to support care planning and the strengthening of public policies for child and adolescent health in Brazil.
